# Integrating the Situational Theory of Problem Solving and Technology Acceptance Model to Predict Intention to Practice Health Protective Behavior for Influenza-Like Illness Among TikTok Users: Cross-Sectional Study

**DOI:** 10.2196/73677

**Published:** 2025-07-02

**Authors:** Can Li, Jen-Sern Tham, Ghazali Akmar Hayati Ahmad, Norliana Hashim, Jeong-Nam Kim

**Affiliations:** 1 Department of Communication Faculty of Modern Languages and Communication Universiti Putra Malaysia Serdang Malaysia; 2 Publicity Department Changzhi University Changzhi China; 3 Brain and Mental Health Research Advancement and Innovation Networks (PUTRA BRAIN) Faculty of Modern Languages and Communication Universiti Putra Malaysia Serdang Malaysia; 4 Moon Soul Graduate School of Future Strategy Korea Advanced Institute of Science and Technology Daejeon Republic of Korea

**Keywords:** health communication, risk perception, social media engagement, behavioral intention, digital health literacy

## Abstract

**Background:**

Outbreaks of influenza-like illness (ILI) pose ongoing public health challenges, prompting widespread demand for timely and accessible health information. TikTok, a leading short video platform, has emerged as an overarching channel for disseminating health-related content, particularly in mainland China. While previous studies have examined health communication on social media, few have integrated complementary theoretical frameworks to understand how user perceptions and motivations jointly influence health behaviors.

**Objective:**

This study integrates the situational theory of problem solving (STOPS) and the technology acceptance model (TAM) to examine the communicative actions and intentions of Chinese TikTok users to adopt health protective behaviors in response to ILI.

**Methods:**

A cross-sectional web-based survey was conducted in China between June and July 2023 using convenience and snowball sampling. A total of 1109 valid responses were analyzed using partial least squares structural equation modeling. Constructs from STOPS (problem recognition, constraint recognition, involvement recognition, situational motivation, and communicative action in problem solving) and TAM (perceived usefulness, perceived ease of use, and attitude) were measured alongside risk perception and intention to engage in protective behaviors.

**Results:**

Perceived usefulness (β=.344; *P*<.001) and ease of use (β=.359; *P*<.001) positively influenced the attitude toward using TikTok. Risk perception (β=.050, *P*=.02) had a small but significant impact on attitude. Situational motivation was positively predicted by risk perception (β=.154; *P*<.001), problem recognition (β=.153; *P*<.001), and involvement recognition (β=.248; *P*<.001) but negatively predicted by constraint recognition (β=−.265; *P*<.001). Both attitude (β=.390; *P*<.001) and situational motivation (β=.471; *P*<.001) significantly influenced communicative action, which in turn predicted intention to practice protective behaviors (β=.570; *P*<.001). Mediation analyses confirmed the partial mediating roles of attitude and situational motivation.

**Conclusions:**

TikTok is an effective platform for public health communication in China, particularly for ILI-related content. Integrating the STOPS and TAM provides a robust framework for explaining how user perceptions and motivations translate into digital engagement and health protective intentions. These findings suggest that interventions should not only enhance technological usability and credibility but also tailor content to elevate perceived personal relevance and reduce psychological or contextual constraints. Future public health campaigns can benefit from engaging influencers, using participatory content formats, and targeting specific motivational cues to increase user involvement in health communication and behavioral change. Caution is warranted in generalizing these results because of the culturally specific and demographically skewed nature of the sample.

## Introduction

### Overview

The global spread of influenza-like illness (ILI) continues to present serious public health challenges. In the age of digital communication and health misinformation, timely, accessible, and accurate health information is crucial [1]. Social media platforms, particularly TikTok, are reshaping how users engage with health content [2]. Thus, there is a need for new theoretical frameworks to understand and guide this behavior. ILI, caused by influenza viruses, presents symptoms such as sudden fever, sore throat, and cough [3]. While often self-limiting in healthy individuals, ILI poses serious risks to vulnerable groups, including children, pregnant women, older adults, and individuals with chronic diseases [4]. Since the early 21st century, China has faced several major outbreaks, including SARS (2003), H1N1 (2009), H7N9 (2013), and COVID-19 (2020) [5]. These epidemics have strained public health systems [6] and heightened public concern, increasing the demand for timely, effective communication to enhance awareness, encourage preventive behaviors, and mitigate ILI impacts [7].

With the rise of mobile and digital technologies, social media platforms have become primary channels for health information dissemination. Platforms such as WeChat [8], Twitter (subsequently rebranded X) [9], and Instagram [10] enable real-time updates, interactive features, and peer-to-peer communication that can rapidly spread public health messages. Compared with traditional mass media, social media supports 2-way communication, increases message reach, and fosters user-generated content, making it an essential tool in modern health campaigns [11,12].

Among these platforms, TikTok has rapidly emerged as a dominant force, especially among younger demographics. In China, the platform boasts >600 million daily active users and is widely used not just for entertainment but also for educational and health-related content [13]. Its short-form video format, algorithmic personalization, and interactive features, such as commenting, sharing, and duets, create a highly engaging environment for health communication [11]. During outbreaks such as the COVID-19 pandemic and seasonal influenza, TikTok became a key channel for disseminating preventive health information [2]. However, despite its popularity and potential, limited research has examined the motivational drivers that, in conjunction with users’ psychological traits and the technological affordances of TikTok, shape their health-related behaviors. Understanding this dynamic is essential for designing effective public health interventions on rapidly evolving digital platforms.

**Figure 1:**
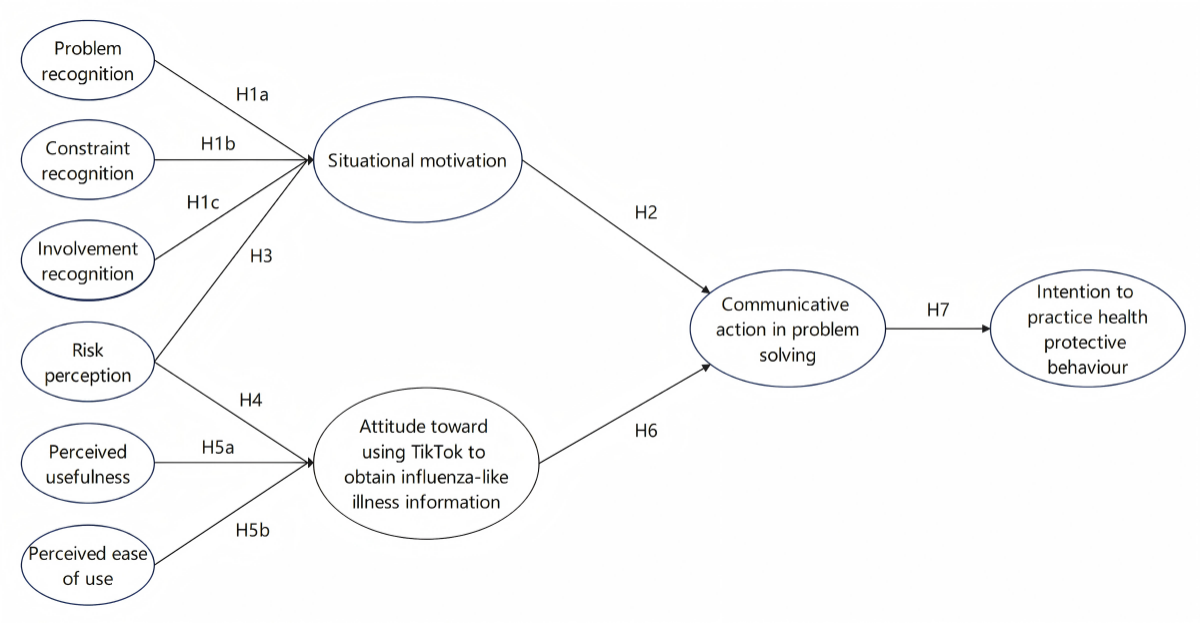
The conceptual framework.

Although existing research has examined either user motivation or technology perceptions in digital health contexts, few studies have integrated both perspectives to explain health communication behaviors on short-form video platforms such as TikTok. This study examines how communicative actions on TikTok influence problem solving behaviors related to ILI. By integrating the situational theory of problem solving (STOPS) and the technology acceptance model (TAM; Figure 1), it offers a more comprehensive theoretical framework than either model alone [14].

The STOPS explains how individuals recognize problems, perceive constraints, and become motivated to seek and share information in response to public issues [15]. The TAM focuses on how users’ beliefs about a technology’s usefulness and ease of use shape their attitudes and adoption behavior [16]. Integrating the STOPS and TAM enables a more comprehensive understanding of digital health engagement by capturing both psychological motivation and technological acceptance. While the STOPS accounts for users’ cognitive appraisal of health threats and their motivation to engage in communicative action, the TAM captures their attitudes toward the technological platform through which such communication occurs. Together, these models explain not just why users are motivated but also how their perception of the platform facilitates or hinders that motivation.

The contributions are threefold: first, by combining problem solving and technology adoption perspectives, this study provides a holistic understanding of health communication behaviors, avoiding the limitations of single-theory approaches [14]. Second, it expands research on health information acquisition and protective behaviors by incorporating risk perception into a communication model. Unlike prior studies focused solely on information seeking [17,18], it examines how situational motivation drives communicative action, shaping health protective intentions. In addition, it highlights how attitudes toward TikTok’s usefulness and ease of use influence engagement with content related to ILI. Third, by investigating the mediating roles of attitude and situational motivation within a Chinese sample, this study enhances the cross-cultural applicability of communicative action models. Identifying how these mediators shape engagement provides deeper insights into the cognitive and motivational mechanisms driving health communication in digital and risk perception contexts. The findings offer theoretical advancements in health communication research and practical implications for public health campaigns, digital health literacy, and targeted interventions on social media platforms.

### Theoretical Background

#### Overview

This study draws upon 2 complementary frameworks: the STOPS and the TAM, to explain how individuals interact with health information on TikTok during ILI outbreaks. In addition, risk perception is incorporated as a psychological antecedent relevant to health behavior. Together, these frameworks provide a multidimensional lens to examine users’ motivations, technological perceptions, and behavioral intentions.

#### STOPS and Research Hypotheses

The STOPS, developed by Kim and Grunig [15], builds on the earlier situational theory of publics to shift the focus from public engagement to individual cognitive and behavioral problem solving through communication. The STOPS conceptualizes individuals as active problem solvers who seek to address issues and their consequences [19]. The theory examines how problem recognition influences communicative behavior in problematic situations. It introduces 4 independent variables—problem recognition, constraint recognition, involvement recognition, and referent criteria—along with situational motivation as a mediator and communicative action as the dependent variable. The STOPS posits that individuals with high problem recognition, greater perceived relevance, and fewer constraints exhibit stronger situational motivation, leading to more active communicative behaviors [15]. Beyond explaining how perceptions of a problem shape communication, the STOPS provides a comprehensive framework for understanding why individuals engage in communicative action when confronted with issues [20].

ILI pandemics trigger varying levels of public concern and engagement, which can be understood through the STOPS framework [21]. At its core, problem recognition refers to individuals’ perception of an issue’s severity. When people recognize ILI as a serious threat, they are more likely to engage in information seeking and communicative behaviors [15]. However, action is also shaped by constraint recognition—perceived barriers that hinder effective responses [21]. Those who feel powerless because of a lack of resources, knowledge, or external support are less likely to engage in problem solving communication. In contrast, involvement recognition determines how personally relevant individuals perceive the issue [22]. Individuals who face personal health risks, family responsibilities, or occupational exposure are more motivated to seek information and take precautions. Situational motivation, or the willingness to learn more about a problem [15], plays a key role. When individuals recognize ILI as a serious and personally relevant issue, they exhibit higher situational motivation, leading to proactive information seeking. Conversely, overwhelming constraints diminish motivation, reducing engagement. Empirical studies confirm these relationships, showing that problem recognition, involvement recognition, and situational motivation positively correlate, while constraint recognition negatively impacts situational motivation [23-25]. These findings provide a strong theoretical basis for analyzing public responses to health crises through communication and information seeking behaviors. Thus, we propose the following first hypotheses:

- Hypothesis 1a: there is a positive relationship between problem recognition and situational motivation.
- Hypothesis 1b: there is a negative relationship between constraint recognition and situational motivation.
- Hypothesis 1c: there is a positive relationship between involvement recognition and situational motivation.

In the STOPS, communicative action in problem solving (CAPS) is the second-order dependent variable, comprising 3 key communicative stages: information acquisition, information selection, and information transmission [20]. Each stage includes active and passive dimensions, resulting in 6 types of communicative actions [15]. At the information acquisition stage, information seeking is an active behavior where individuals intentionally search for ILI-related content on TikTok, while information attending is a passive behavior involving incidental exposure [26]. The information selection stage differentiates between information forefending—actively rejecting misleading content—and information permitting, where individuals accept all information without scrutiny [22]. The information transmission stage includes information forwarding, where users actively share ILI-related content, and information sharing, where they pass along content upon request [15]. Notably, active behaviors encompass passive ones but not vice versa [19]. This framework provides a nuanced understanding of how individuals process and disseminate health information.

The STOPS identifies situational motivation as a key predictor of communicative action [27]. Individuals with higher situational motivation engage more actively in communication. Empirical studies support this across various contexts: for instance, the study by Krishna and Amazeen [28] linked situational motivation to active vaccine-related communication, while the study by Lee et al [29] showed it drove antiracism mobilization during the COVID-19 pandemic. The study by Yan et al [30] found that situational motivation is positively associated with seeking, forefending, and forwarding diet-related information on the web. These findings highlight its pivotal role in shaping positive communication behaviors on social media. Thus, this study hypothesizes as follows:

- Hypothesis 2: there is a positive relationship between situational motivation and CAPS.

#### The Role of Risk Perception

While the STOPS provides a robust framework for understanding how individuals identify and respond to problems through communication, it does not fully account for cognitive-emotional responses, such as perceived risk, which is especially critical in health contexts. Therefore, this study incorporates risk perception as a key antecedent influencing communication motivation.

Originating in psychology as a feeling of uncertainty [31], risk perception has become a critical variable in health communication research [32,33]. It is a key predictor in major theoretical frameworks, including the health belief model [34], protection motivation theory [35], and the extended parallel process model [36], underscoring its significance in understanding health behaviors [37]. Risk perception refers to individuals’ subjective assessment of potential risks and is measured through affective, cognitive, and spatiotemporal dimensions [38]. In China, regional differences make the spatiotemporal aspect particularly influential. When individuals perceive health risks, they often experience negative emotions such as worry or fear [39], prompting them to seek information and adopt health protective behaviors to mitigate anxiety. Empirical studies confirm its predictive role in health communication. The study by Yan et al [30] integrated the STOPS and risk perception, demonstrating its positive impact on health information engagement. Similarly, the study by Liu et al [40] found that heightened risk perception during public health emergencies increased situational motivation for health information behaviors. These findings reinforce the role of risk perception in shaping health communication and behavioral intentions. Therefore, the following hypothesis is proposed:

- Hypothesis 3: there is a positive relationship between risk perception and situational motivation.

In the context of ILI, risk perception drives individuals’ need for information, prompting them to use social media platforms such as TikTok to seek relevant content [30]. TikTok provides diverse health resources that cater to users’ information needs [2]. When individuals perceive a higher personal and public health risk, they are more likely to turn to TikTok for information. Research confirms that risk perception influences information seeking behaviors [41]. For instance, the study by Basch et al [2] found that individuals use various channels to manage health risks. Similarly, studies show that health care professionals’ COVID-19 risk perception influenced precautionary behaviors in Ethiopian hospitals [42], while the study by Guo et al [43] found that risk perception significantly predicts cardiovascular health behaviors. These findings reinforce the role of risk perception in shaping digital health engagement. On the basis of these findings, the following hypothesis is proposed:

- Hypothesis 4: there is a positive relationship between risk perception and attitude toward using TikTok to obtain ILI information.

#### TAM and Research Hypotheses

Although risk perception helps explain user motivation, it does not account for perceptions of the technological platform through which communication occurs. In digital environments, the decision to engage with health content is also influenced by how users perceive the platform [44]. The TAM is a leading framework in information systems research, widely used to examine technology adoption and implementation [45,46]. It has been extensively applied in health research to understand the adoption of health-related technologies, including social media [47]. Using a belief-attitude-intention-behavior framework, the TAM provides insights into how users acquire and share health information. The TAM posits that user adoption is driven by technological beliefs, particularly perceived usefulness and perceived ease of use [16]. Empirical studies support this: the study by Rajak and Shaw [48] found that these factors positively influence attitudes toward mobile health technology. Similarly, the study by Kahil and Alobidyeen [11] demonstrated that Dutch users’ perceptions of TikTok’s usefulness and ease of use during the COVID-19 pandemic positively influenced their adoption of procedural learning. These findings underscore TAM’s relevance in understanding social media’s role in health information dissemination. Accordingly, this study proposes the following hypotheses:

- Hypothesis 5a: there is a positive relationship between perceived usefulness and attitude toward using TikTok to obtain ILI information.
- Hypothesis 5b: there is a positive relationship between perceived ease of use and attitude toward using TikTok to obtain ILI information.

#### Integrated STOPS and TAM

Taken alone, the STOPS emphasizes problem- and motivation-related variables, while the TAM focuses on technology-related attitudes. However, these models can be integrated to holistically explain user engagement on TikTok. The next section outlines how combining the STOPS and TAM offers a richer understanding of digital health communication.

While the STOPS effectively explains how individuals recognize problems and engage in communicative actions, it does not fully account for the role of technology in shaping behavior. In digital environments, information seeking is influenced not only by situational motivation but also by perceptions of technology’s usefulness and ease of use. Given TikTok’s role in health information exchange, integrating the TAM enhances STOPS’ explanatory power in digital contexts [14]. The TAM complements the STOPS by incorporating perceived usefulness and perceived ease of use as key determinants of technology adoption. This integration extends the STOPS beyond traditional problem solving by considering how technological perceptions shape communicative behaviors in health risk contexts. Empirical research supports this approach: positive attitudes toward digital platforms enhance communicative engagement. For instance, the study by Zheng [14] found that attitudes toward mobile phone donation influenced communication behaviors, while the study by Zagidullin et al [49] demonstrated that trust in social media fosters engagement and content sharing. Similarly, the study by Lee et al [50] revealed that positive attitudes toward social media advertising correlated with increased factual information sharing. These findings reinforce the importance of integrating the TAM with the STOPS to better understand health communication on digital platforms such as TikTok. Building on these findings, this study proposes the following hypothesis:

- Hypothesis 6: there is a positive relationship between attitude toward using TikTok and CAPS.

#### Extending the STOPS With the Inclusion of Intention to Practice Health Protective Behavior

Although communicative actions are key to digital engagement, the ultimate goal of public health communication is behavioral change. Thus, we extend the integrated model by adding health protective intention as the outcome variable. Beyond predicting communication behaviors, the STOPS can be extended to forecast individuals’ intentions to adopt health protective behaviors, defined as the willingness to engage in self-protective actions to mitigate ILI risk during epidemics [51]. These measures—vaccination, handwashing, mask-wearing, social distancing, and avoiding contact with infected individuals—reduce individual susceptibility and contribute to public health stability [52]. Extending the STOPS to behavioral intentions aligns with the broader goal of health communication to promote preventive actions. Prior research supports this extension, as the STOPS has been applied to health-related decision-making processes [20,22]. Positioning health protective intentions as an outcome variable provides a more holistic perspective on how individuals transition from communicative actions to preventive behaviors during health crises. On the basis of these theoretical foundations, this study proposes the following hypothesis:

- Hypothesis 7: there is a positive relationship between CAPS and the intention to practice health protective behavior.

#### Attitude and Situational Motivation as Mediators

Beyond direct effects, the mechanisms through which situational and technological factors influence behavior require further exploration. In this study, we focus on 2 key mediators—attitude and situational motivation—that explain how perceptions translate into action [15]. As a cognitive and affective driver, situational motivation explains why individuals engage in specific information behaviors when confronted with a problem. Empirical studies confirm this mediating role across various domains, including COVID-19 pandemic responses [4], public health crisis communication [22], and fraud risk assessment [25]. Given its broad applicability, this study proposes the following:

- Hypothesis 8: situational motivation mediates the effect of (1) problem recognition, (2) constraint recognition, (3) involvement recognition, and (4) risk perception on CAPS.

Similarly, attitude plays a dual role in TAM. The study by Davis [16] identified its mediating function in technology adoption, though empirical findings on its role remain inconsistent. Some studies support its mediating role between beliefs and behavioral intention [53,54], while others contest it [55]. Scholars argue that dismissing attitudes without thorough theoretical consideration limits understanding, calling for reassessment [56]. Therefore, this study proposes the following:

- Hypothesis 9: attitude toward using TikTok for ILI information mediates the effect of (1) perceived usefulness and (2) perceived ease of use on CAPS.

## Methods

### Sample and Data Collection

To ensure that all respondents evaluated the same version and features of the TikTok platform, this study applied three inclusion criteria: (1) active use of TikTok, (2) residency in mainland China, and (3) age ≥18 years. These criteria were necessary because the platform’s features and functionalities differ for users <18 years and those in Hong Kong, Macau, and Taiwan compared with mainland China.

All questionnaire items were adapted from a previous study. Because this study was conducted in China, the authors translated the questionnaire into Chinese. To ensure accuracy and clarity of the translation, the questionnaire was reviewed by 3 bilingual experts in communication studies—1 from Malaysia and 2 from China. The experts provided feedback on linguistic accuracy and conceptual consistency, leading to revisions that enhanced the clarity and validity of the instrument. Following the revisions, a pilot test was conducted. The results indicated satisfactory internal consistency, with Cronbach α coefficients exceeding 0.7 for all measured variables.

Because TikTok has >600 million daily active users in China [13], implementing probability sampling would be impractical. Therefore, this study used nonprobability sampling methods, specifically snowball and convenience sampling. A cross-sectional survey was conducted in China between June and July 2023. A total of 1590 respondents completed the questionnaires. After excluding ineligible responses (ie, those who failed to answer the attention-check question or who finished the entire survey within 5 min), 1109 valid questionnaires were retained for analysis, yielding a response rate of 70%. Among the respondents, female participants (620/1109, 55.9%) outnumbered male participants (Table 1). The sample was relatively young, with an average age of 30.6 (SD 8.43) years and a median age of 30 years. The majority had a high level of educational attainment, with 79.8% (885/1109) holding a bachelor’s degree or higher. In terms of monthly income, more than 50% (602/1109, 54.28%) fell into the middle-income or higher category (monthly income >5000 CNY=US $700).

**Table 1:** Demographic description (N=1109).

Characteristics		Values
Age (y), mean (SD)		30.69 (8.43)
**Sex, n (%)**		
	Male	489 (44.1)
	Female	620 (55.9)
**Education, n (%)**		
	No schooling	0 (0)
	Primary school	3 (0.3)
	Junior secondary school	9 (0.8)
	Senior secondary school	69 (6.2)
	Medium vocational education	34 (3.1)
	High vocational education	10 (0.9)
	College	99 (8.9)
	University	803 (72.4)
	Graduate higher level	82 (7.4)
**Income (CNY^a^), n (%)**		
	No income	168 (15.1)
	<500	23 (2.1)
	501-1000	20 (1.8)
	1001-1500	36 (3.2)
	1501-2000	29 (2.6)
	2001-3000	38 (3.4)
	3001-5000	193 (17.4)
	5001-8000	294 (26.5)
	>8000	308 (27.8)

^a^The average conversion rate during the study was 1 CNY=US $0.14.

### Measurements

All measurement scales in the questionnaire, except for the attitude scale, which used 6-point semantic differential pairs, used a 6-point scale without a neutral midpoint. Specifically, a 4-item scale, adapted from the study by Kim and Grunig [15], was used to measure *problem recognition* (α=0.718; mean 4.32, SD 0.32). To measure *constraint recognition*, we referred to the studies by Kim and Grunig [15], Chon et al [22], and Lee et al [57] (α=0.866; mean 3.06, SD 0.94). *Involvement recognition* was measured using a 4-item scale, adapted from the studies by Kim and Grunig [15] and Liu et al [58] (α=0.767; mean 4.48, SD 0.79). We referred to the studies by Kim and Grunig [15] to measure *situational motivation* (α=0.722; mean 3.90, SD 0.95). In addition, both *perceived usefulness* (α=0.805; mean 4.50, SD 0.83) and *perceived ease of use* (α=0.728; mean 4.65, SD 0.81) were measured using a 3-item scale, adapted from the study by Rajak and Shaw [48]. To assess *risk perception*, we adapted 3 dimensions (a total of 6 items) from the study by Dryhurst et al [38] (α=0.857; mean 5.05, SD 0.59). Moreover, 9 items were adapted from the study by Mao and Hovick [59] to assess *attitude toward using TikTok* to obtain ILI information (α=0.947; mean 4.55, SD 0.90). Furthermore, c*ommunicative action in problem solving* has been advised to be modeled as a reflective–reflective second-order construct [15] (α=0.931; mean 4.29, SD 0.80). A total of 24 items, adapted from the study by Kim and Grunig [15], cover 6 interrelated first-order constructs: *information seekin*g (α=0.871; mean 4.20, SD 0.99), *information attending* (α=0.847; mean 4.21, SD 0.92), *information forefending* (α=0.750; mean 4.76, SD 0.76), *information permitting* (α=0.797; mean 3.90, SD 0.92), *information forwarding* (α=0.862; mean 4.02, SD 1.02), and *information sharing* (α=0.806; mean 4.15, SD 0.93). Finally, *intention to practice health protective behavior* was measured using a 6-item scale adopted from the study by Ho et al [60] (α=0.817; mean 4.42, SD 0.92). The detailed items are presented in Multimedia Appendix 1.

### Ethical Considerations

This study obtained ethics approval from Changzhi Medical College (RT2023046). Before participation, all respondents were informed about the study’s purpose, procedures, and rights as participants. Informed consent was obtained, ensuring that participation was voluntary and that respondents could withdraw at any time without any consequences. Participants were also assured that their responses would remain confidential and be used solely for research purposes. No personally identifiable information was collected, and all data were handled in accordance with ethical guidelines to protect participants’ privacy and rights. All eligible participants who completed the survey were allocated randomized monetary compensation of 2-5 CNY (US $0.28-$0.70), disbursed electronically immediately following survey completion.

### Data Analysis Approach

Partial least squares structural equation modeling (PLS-SEM) was chosen as the primary analytic method, using SmartPLS (version 4.0; SmartPLS GmbH). Several reasons supported this choice. First, this study was fundamentally exploratory, involving an extension of the theoretical models, the STOPS and TAM. Second, the normality of the dependent variable was tested using both the Kolmogorov-Smirnov and Shapiro-Wilk tests. Both tests returned statistically significant results (*P*<.001), indicating that the variables did not follow a normal distribution, which limits the suitability of covariance-based structural equation modeling [61,62]. Third, the hypothesized model is complex, encompassing 10 latent variables (66 observed variables), including a second-order variable. PLS-SEM demonstrates advantages in handling complex models efficiently [63].

## Results

The proposed model tested in this study evaluates relationships between situational motivation, risk perception, technological beliefs, communicative action, and behavioral intention. These relationships are grounded in the STOPS and the TAM.

### Measurement Model Analysis

Following the statistical analysis guidelines [64,65], we assessed the measurement model by evaluating Cronbach α, composite reliability (CR), indicator loadings, and average variance extracted (AVE). The acceptable thresholds are as follows: Cronbach α and CR should be ≥0.7, and indicator loadings and AVE should be ≥0.5. The item IFF4 had a low loading value of 0.30, which was below the acceptable threshold for indicator reliability. Consequently, it was deleted to improve the overall model fit. After the deletion, Cronbach α for information forefending increased to 0.75, the CR value rose to 0.77, and the AVE value increased to 0.66, all surpassing the recommended thresholds. The Cronbach α values for all variables ranged from 0.71 (problem recognition) to 0.95 (attitude toward using TikTok to obtain ILI information). Similarly, the CR values fell within the range of 0.72 (situational motivation) to 0.93 (attitude toward using TikTok to obtain ILI information), with all values exceeding the recommended threshold of 0.7. Furthermore, the AVE values ranged from 0.52 (intention to practice health protective behavior) to 0.74 (CAPS), while all indicator loadings ranged from 0.59 (PR1) to 0.87 (CR2), with both metrics surpassing the suggested threshold of 0.50. The detailed results for all variables are presented in Multimedia Appendix 1.

Testing discriminant validity is also a crucial step. The heterotrait-monotrait ratio is used to assess the degree of similarity between two constructs; a ratio above the threshold indicates potential issues with discriminant validity. According to the study by Henseler et al [66], acceptable heterotrait-monotrait values should be <0.85 or 0.9, with the more stringent threshold of 0.85 applied in this study. As shown in Table 2, the results confirm strong discriminant validity among the 10 variables. The correlation coefficients for each pair of variables remained consistently low, all <0.85, indicating minimal intercorrelation and supporting the independence of the variables. This strengthens the reliability of the measurement tool, ensuring that each variable’s influence can be accurately assessed. We also considered variance inflation factors (VIF). Reporting VIF values is a standard practice for assessing collinearity [63]. According to the study by Kock [67], VIF should be ≤3.3 to ensure no bias in single-source data. In this study, the VIF for all constructs ranged from 1.13 to 3.24, indicating that VIF was not a concern.

**Table 2:** Heterotrait-monotrait ratio for discriminant validity.

	ATT^a^	IPH^b^	CAPS^c^	CR^d^	IR^e^	PEOU^f^	PR^g^	PU^h^	RP^i^	SM^j^
ATT	—^k^	—	—	—	—	—	—	—	—	—
IPH	0.348	—	—	—	—	—	—	—	—	—
CAPS	0.543	0.64	—	—	—	—	—	—	—	—
CR	0.323	0.434	0.658	—	—	—	—	—	—	—
1R	0.339	0.452	0.541	0.401	—	—	—	—	—	—
PEOU	0.692	0.371	0.566	0.304	0.419	—	—	—	—	—
PR	0.4	0.631	0.714	0.553	0.83	0.435	—	—	—	—
PU	0.663	0.438	0.637	0.414	0.375	0.805	0.495	—	—	—
RP	0.204	0.415	0.489	0.311	0.639	0.206	0.685	0.269	—	—
SM	0.309	0.417	0.696	0.579	0.679	0.292	0.698	0.412	0.554	—

^a^ATT: attitude toward using TikTok to obtain influenza-like illness information.

^b^IPH: Intention to practice health protective behavior.

^c^CAPS: communicative action in problem solving.

^d^CR: constraint recognition.

^e^IR: involvement recognition.

^f^PEOU: perceived ease of use.

^g^PR: problem recognition.

^h^PU: perceived usefulness.

^i^RP: risk perception.

^j^SM: situational motivation.

^k^Not applicable.

### Structural Model Analysis

**Figure 2:**
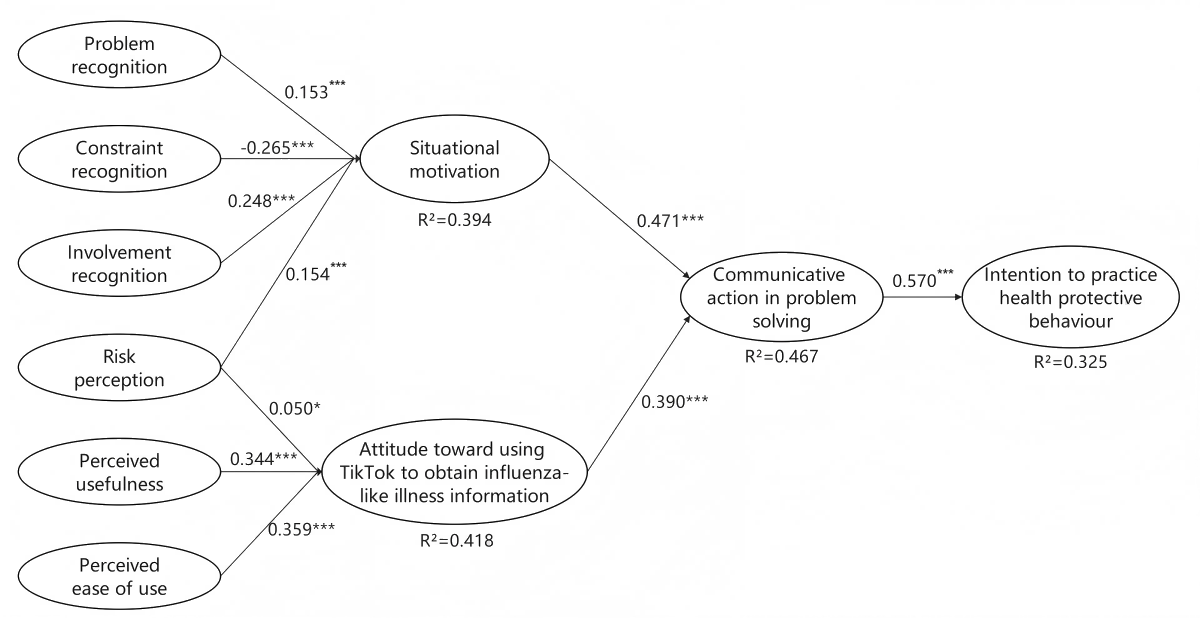
Structural model. *P<.05, ***P<.001.

The assessment of the significance of path coefficient in PLS-SEM heavily relies on the bootstrapping method, with a significance level of 95% [64]. This study used bootstrapping with 5000 subsamples using SmartPLS to analyze the path coefficients. The structural model diagram is shown in Figure 2.

The results (Table 3) indicated positive associations between problem recognition (β=.153, *t*=3.742; *P*<.001) and involvement recognition (β=.248, *t*=6.658; *P*<.001) with situational motivation, while constraint recognition showed a significant negative relationship (β=−.265, *t*=8.224; *P*<.001), thus supporting hypothesis 1a, hypothesis 1b and hypothesis 1c. A significant positive association was found between situational motivation and communicative action in the problem (β=.471, *t*=18.543; *P*<.001), supporting hypothesis 2. In addition, risk perception was positively associated with situational motivation (β=.154, *t*=4.516; *P*<.001), supporting hypothesis 3. A positive and significant relationship was observed between risk perception and attitude toward using TikTok to obtain ILI information (β=.050, *t*=4.516; *P*=.02), supporting hypothesis 4. Perceived usefulness (β=.344, *t*=10.404; *P*<.001) and perceived ease of use (β=.359, *t*=11.106; *P*<.001) were significant positive predictors of attitude, supporting hypothesis 5. Furthermore, attitude toward using TikTok to obtain ILI information was positively related to communicative action in the problem solving (β=.390, *t*=14.911; *P*<.001), supporting hypothesis 6. Finally, communicative action in the problem solving significantly predicted the intention to practice health protective behavior (β=.570, *t*=23.323; *P*<.001), supporting hypothesis 7.

**Table 3:** Assessment of the path coefficient of direct relationship (N=1109).

Hypothesis	Relationship	Original sample, β coefficient	Sample mean (SD)	*t* test	*P* value	Decision
Hypothesis 1a	PR^a^→SM^b^	.153	0.154 (0.041)	3.742	<.001	Supported
Hypothesis 1b	CR^c^→SM	−.265	−0.266 (0.032)	8.224	<.001	Supported
Hypothesis 1c	IR^d^→SM	.248	0.247 (0.037)	6.658	<.001	Supported
Hypothesis 2	SM→CAPS^e^	.471	0.471 (0.025)	18.543	<.001	Supported
Hypothesis 3	RP^f^→SM	.154	0.154 (0.034)	4.516	<.001	Supported
Hypothesis 4	RP→ATT^g^	.050	0.050 (0.021)	2.360	.02	Supported
Hypothesis 5a	PU^h^→ATT	.344	0.345 (0.033)	10.404	<.001	Supported
Hypothesis 5b	PEOU^i^→ATT	.359	0.359 (0.032)	11.106	<.001	Supported
Hypothesis 6	ATT→CAPS	.390	0.391 (0.026)	14.911	<.001	Supported
Hypothesis 7	CAPS→IPH^j^	.570	0.571 (0.024)	23.323	<.001	Supported

^a^PR: problem recognition.

^b^SM: situational motivation.

^c^CR: constraint recognition.

^d^IR: involvement recognition.

^e^CAPS: communicative action in problem solving.

^f^RP: risk perception.

^g^ATT: attitude toward using TikTok to obtain influenza-like illness information.

^h^PU: perceived usefulness.

^i^PEOU: perceived ease of use.

^j^IPH: intention to practice health protective behavior.

### Test of Hypotheses in Mediation Effect

SPSS (version 27) with PROCESS (version 4.0) was used to analyze the mediating effects, using 5000 bootstrap samples to estimate SEs.

The results (Table 4) revealed the direct effects of problem recognition, constraint recognition, involvement recognition, and risk perception on CAPS to be 0.4244, −0.3522, 0.2366, and 0.2161, respectively. Situational motivation positively mediated the relationship between problem recognition (β=.1916, 95% CI 0.1565-0.2301), involvement recognition (β=.2288, 95% CI 0.1893-0.2697), risk perception (β=.1845, 95% CI 0.1514-0.2208), and CAPS. Conversely, situational motivation negatively mediated the relationship between constraint recognition and CAPS (β=−.1449, 95% CI −0.1738 to −0.1172). These findings support hypothesis 8, indicating that situational motivation acts as a partial mediator. The results also showed that the direct effects of perceived usefulness and perceived ease of use on CAPS were 0.3754 and 0.2456, respectively. The attitude toward using TikTok to obtain ILI information positively mediated the relationships between perceived usefulness (β=.1592, 95% CI 0.1181-0.2046), perceived ease of use (β=.2075, 95% CI 0.1599-0.2625), and CAPS. Thus, hypothesis 9 was supported. These findings suggest that attitude toward using TikTok to obtain ILI information functions as a partial mediator.

**Table 4:** Mediation analysis results at 95% CI.

Dependent variable: CAPS^a^	Direct effect, β (95% CI)	Indirect effect, β (95% CI)
PR^b^→SM^c^→CAPS	0.4244 (0.3708 to 0.4780)	.1916 (0.1565 to 0.2301)
CR^d^→SM→CAPS	−0.3522 (−0.3936 to −0.3109)	−.1449 (−0.1738 to −0.1172
IR^e^→SM→CAPS	0.2366 (.1812 to 0.2921)	.2288 (0.1893 to 0.2697)
RP^f^→SM→CAPS	0.2161 (0.1682 to 0.2639)	.1845 (0.1514 to 0.2208)
PU^g^→ATT^h^→CAPS	0.3754 (0.3192 to 0.4316)	.1592 (0.1181 to 0.2046)
PEOU^i^→ATT→CAPS	0.2456 (0.1859 to 0.3052)	.2075 (0.1599 to 0.2625)

^a^CAPS: communicative action in problem solving.

^b^PR: problem recognition.

^c^SM: situational motivation.

^d^CR: constraint recognition.

^e^IR: involvement recognition.

^f^RP: risk perception.

^g^PU: perceived usefulness.

^h^ATT: attitude toward using TikTok to obtain influenza-like illness information.

^i^PEOU: perceived ease of use.

### Coefficient of Determination, Effect Size, and Predictive Relevance

In addition to testing hypotheses, the structural model also assessed the coefficient of determination (*R*^2^), effect size (f*^2^*), and predictive relevance (*Q*^2^) [63] The study by Hair et al [64] asserted that *R*^2^ values of 0.25, 0.50, and 0.75 are categorized as weak, moderate, and substantial, respectively. Our model explained 0.394 of the variance (*R*^2^) in situational motivation, 0.418 in attitude toward using TikTok to obtain ILI information, 0.467 in CAPS, and 0.325 in intention to practice health protective behavior. As mentioned earlier, according to the criteria of *R*^2^ value proposed by Hair et al [63], these *R*^2^ values indicate weak-to-moderate levels of predictive accuracy.

On the basis of Cohen [68] criteria, the f² values of 0.121 for perceived usefulness and 0.135 for perceived ease of use indicate small effect sizes in generating *R*² for attitude toward using TikTok for ILI information. The effect size between risk perception and attitude, with Cohen f²=0.004, is considered extremely small, far below the threshold for a small effect (f²=0.02), indicating minimal practical significance. Attitude toward using TikTok has a medium effect size (f²=0.268) in producing *R*² for CAPS, while situational motivation has a large effect size (f²=0.390) for the same outcome. Risk perception (f²=0.025), constraint recognition (f²=0.092), and involvement recognition (f²=0.057) show small effect sizes in generating *R*² for situational motivation, while problem recognition (f²=0.019) is considered very small. Finally, CAPS shows a small effect size (f²=0.482) in generating *R*² for the intention to practice health protective behaviors.

This study also confirmed the predictive accuracy (*Q*^2^) of our proposed research framework, where *Q*^2^ values above 0.50, 0.25, and 0 depict large, medium, and small predictive accuracy, respectively [64]. This study calculated the highest *Q*^2^ value for CAPS at 0.345. Conversely, the lowest was calculated for the intention to practice health protective behaviors at 0.163. All *Q*^2^ values for the endogenous latent variables were >0, indicating that the model exhibits a satisfactory level of predictive ability.

## Discussion

### Principal Findings

ILI-related information is among TikTok’s most frequently searched health topics [69]. Users engage by uploading, searching, sharing, liking, and commenting on related content, yet research lacks insight into the motivations behind these behaviors. This study integrates the STOPS and the TAM to predict TikTok users’ communicative actions and health protective intentions regarding ILI in mainland China. By incorporating risk perception as a key antecedent influencing attitude and situational motivation, this study extends existing models to better explain digital health communication. The results support most hypothesized relationships, showing that users’ problem recognition, involvement recognition, technological attitudes, and motivation significantly predict communicative actions, which in turn predict health protective intentions.

The findings extend the applicability of the STOPS and TAM by demonstrating how they operate together in a short video platform environment. They emphasize that both psychological triggers (eg, situational motivation) and technological perceptions (eg, perceived usefulness) jointly influence engagement. In the following sections, we interpret these results in relation to previous literature and practical application.

#### Risk Perception and Situational Recognition of Health Motivation

This study demonstrates that problem recognition and involvement recognition positively influence situational motivation to address ILI-related issues, while constraint recognition significantly negatively affects situational motivation. These findings align with the core assumptions of the STOPS and are consistent with prior health communication research [15,22,27,40]. However, the impact of problem recognition on situational motivation appears relatively limited. One explanation may lie in the perceived nature of ILI itself. Unlike severe public health crises, such as COVID-19 or avian influenza, ILI is generally seen as a manageable and routine condition. Symptoms are mild, and recovery is often straightforward with home remedies or basic medical care [3]. Consequently, users may cognitively acknowledge the existence of ILI but not perceive it as an urgent issue requiring problem solving behavior. This aligns with research showing that individuals prioritize information seeking behaviors for conditions perceived as novel, severe, or life threatening [40]. For example, during emerging pandemics, public anxiety and uncertainty amplify risk perception and engagement; in contrast, the familiar nature of ILI reduces motivational arousal. Furthermore, the convergence of ILI and COVID-19 symptoms in early 2023 in China and intense media coverage may have created a saturation effect [70]. The sheer volume of ILI-related discourse across news and social media may have reduced individual differentiation in problem perception, thereby weakening its motivational role. This supports the notion that salience alone is insufficient to drive engagement unless combined with personal relevance and emotional urgency.

#### Risk Perception, Technological Beliefs, and User Attitudes on TikTok

Although risk perception significantly influenced situational motivation, it had only a marginal effect on attitudes toward using TikTok for health information. Instead, technological beliefs, perceived usefulness, and ease of use played a more substantial role. This finding underscores a recurring theme in digital behavior research: platform-specific features often override the content context in shaping user attitudes [48]. There are several reasons for the weak influence of risk perception on platform attitude. First, as discussed, ILI does not typically evoke strong affective responses. Without a sense of crisis or high personal threat, individuals may not feel compelled to rely on nontraditional sources such as TikTok. Second, TikTok is still largely viewed as an entertainment-oriented platform. While it hosts increasing amounts of health-related content, users may not see it as a credible or preferred source for serious health information [71]. In fact, higher levels of health-related risk perception may lead users to seek information from more authoritative channels, such as governmental health portals or hospital websites. Studies have shown that increased risk perception can trigger greater scrutiny of source credibility, particularly on platforms primarily known for entertainment [12,72]. Therefore, when health threats are perceived as serious, users may consciously avoid user-generated content in favor of expert-driven messaging.

#### Attitudes Toward TikTok and Communicative Actions

Consistent with the core tenet of the TAM [14,50], this study confirms that a positive attitude toward TikTok significantly predicts communicative actions related to ILI content. Users who find TikTok useful and easy to use are more likely to seek information, engage with health posts, and participate in content dissemination. These behaviors include liking, sharing, and commenting on videos and searching for health tips, prevention methods, and user experiences [14]. Moreover, users with favorable attitudes toward TikTok are more likely to use its interactive affordances, such as duets, challenges, or stitched videos, to share their perspectives or raise awareness. These participatory behaviors contribute to a more vibrant digital health ecosystem, where community dialogue supports both social learning and behavior normalization [73]. Thus, platform perception does not just influence engagement; it actively shapes the health communication environment on TikTok.

#### Mediating Role of Attitude and Situational Motivation

This study also demonstrates that attitude and situational motivation are important mediators in the model. Users who perceive TikTok as a useful and easy-to-navigate platform tend to develop positive attitudes, encouraging them to engage in communicative behaviors. Similarly, individuals who recognize a health problem, feel personally involved, and perceive few constraints are more likely to be motivated to engage. Interestingly, the mediating effect of situational motivation was somewhat weaker than expected compared with earlier STOPS research [15]. One explanation is that users’ behavior on TikTok may be less intentional and more algorithm driven. Exposure to health content is often passive, determined by trending topics or recommended videos rather than deliberate search behaviors [74]. Therefore, even if users are motivated, their actions may be shaped more by platform dynamics than internal cognitive assessments. Because not all TikTok users actively seek health-related content, situational motivation may be influenced by external factors such as trending topics, peer influence, and the availability of credible content. Future research should explore how different health issues and social media environments impact the mediating role of situational motivation in digital health communication behaviors. Future research should also explore how content personalization, algorithmic exposure, and platform norms interact with psychological motivations. For example, do users engage more actively when health content aligns with trending hashtags, or when it is posted by influencers they already follow? These questions can help refine the role of situational motivation in algorithmic environments.

#### Communicative Actions and Protective Behavior Intention

A significant and meaningful finding of this study is the strong relationship between communicative action and health protective intention. Users who engage in seeking, sharing, and discussing ILI-related content are more likely to report an intention to adopt protective measures such as vaccination, mask-wearing, and hygiene practices. This supports existing literature on the power of social media to facilitate health behavior change by promoting observational learning, enhancing perceived behavioral control, and reinforcing social norms [2,12,75]. Engaging in communicative acts likely facilitates deeper cognitive elaboration of health messages, increases perceived self-efficacy, and creates a sense of collective responsibility for health protection. Moreover, interactive communication fosters collective efficacy, wherein seeing others engage in similar health behaviors increases one’s own intention to act. This is particularly relevant on platforms such as TikTok, where content virality, repetition, and peer participation can significantly amplify message salience and behavioral modeling.

### Implications for Research and Practice

This study offers significant contributions to the STOPS, the TAM, and public health practice by integrating both models to examine health information engagement on TikTok and its impact on health protective intentions among Chinese adults. First, this study extends STOPS by confirming that risk perception and situational recognition shape situational motivation, influencing communicative actions and health protective intentions in digital environments. It bridges technological engagement and health problem solving behaviors, highlighting the role of attitude and situational motivation in driving CAPS and subsequent health behaviors. Second, integrating the TAM into health communication research underscores how perceived usefulness and ease of use shape user attitudes, influencing health information–seeking and -sharing behaviors on TikTok. Future research should explore how digital affordances (eg, algorithmic recommendations, interactivity, and personalization) interact with cognitive and motivational states in shaping health information behaviors. Third, the study reinforces attitude and situational motivation as mediators in health communication. Researchers should move beyond direct-effect models, examining these variables across different health contexts, media platforms, and cultural settings to validate their role in digital health engagement.

The findings provide insights for health care professionals, public health policy makers, and digital platform operators in designing effective health communication strategies on TikTok. Given that technology beliefs influence engagement, content should be user-friendly, interactive, and visually appealing (eg, live Q&A, short videos, and influencer collaborations). Because risk perception alone does not strongly drive engagement, public health campaigns should enhance personal involvement and relevance, making ILI threats more personally salient rather than relying on general warnings. Credibility concerns should be addressed through expert collaborations, fact-checking, and verified content to increase trust in TikTok as a health information source. Finally, because active engagement (seeking, sharing, and discussing) on TikTok predicts health protective behaviors, campaigns should prioritize interactive discussions over passive exposure, leveraging challenges, prompts, and community engagement to encourage preventive actions.

### Limitations and Directions for Future Research

Although this study has made significant contributions, its limitations should be acknowledged and addressed in future research. First, this study focuses on the TikTok platform to investigate users’ ILI-related communicative behaviors. The findings must be comprehended within the sociopolitical and cultural context of mainland China. Factors such as government regulation, platform censorship, and varying levels of media trust may uniquely shape user behaviors. These contextual differences may limit the international applicability of the findings. Future research should replicate and extend this study in different cultural and regulatory contexts (eg, liberal democracies, countries with decentralized health systems, or varying levels of digital infrastructure) to assess the universality of the STOPS-TAM integration model. Comparative cross-national studies may help clarify how cultural norms, media freedom, and trust in government or platforms moderate the relationships observed in this study. Such insights will enhance the global applicability of digital health communication frameworks and tailor public health interventions more effectively across sociocultural settings. Second, this study is limited to the discussion of ILI. Future studies should consider other diseases to gain a more comprehensive understanding of the factors influencing health information dissemination. Third, this study is confined to survey respondents from China. Previous studies have shown that the STOPS may affect populations in different countries [14,76]. Therefore, future studies should replicate this model in other countries to conduct in-depth studies on situational motivations in different cultural and social contexts and compare the research results to enhance the generalizability and adaptability of the model. Fourth, this study only used a cross-sectional research design. The nature of a cross-sectional design prohibits drawing causal inferences or examining temporal associations between variables, whether in the model’s stepwise operation or bidirectional. Future research should consider using experimental and longitudinal designs to track the evolution and trends of user behavior. Fifth, the sample in this study was predominantly young, female, and well-educated. As no statistical weighting was applied to adjust for demographic imbalance, generalizability may be limited. These characteristics may influence perceptions of TikTok, digital literacy, and health behaviors, and thus should be interpreted with caution.

### Conclusions

This study contributes to a deeper understanding of how Chinese TikTok users engage with ILI-related health information by integrating the STOPS and the TAM. Using a PLS-SEM approach, we empirically analyzed the relationships among cognitive, motivational, and technological constructs that influence communicative actions and health protective behavioral intentions. Our findings show that perceived usefulness and ease of use significantly influence users’ attitudes toward using TikTok, while problem recognition, constraint recognition, and involvement recognition shape situational motivation. Risk perception plays a dual role by affecting both attitude and motivation. Furthermore, both attitude and situational motivation predict CAPS, which in turn strongly influences users’ intention to engage in health protective behaviors. Notably, attitude and situational motivation serve as partial mediators, offering a more nuanced understanding of how digital engagement leads to behavioral outcomes. These findings have several practical implications for health communication strategies and public health interventions on social media platforms such as TikTok. Public health agencies and content creators should focus on designing campaigns that are both technologically accessible and emotionally engaging. This includes using TikTok-native formats such as short videos, interactive challenges, and influencer collaborations to increase perceived usefulness and user motivation. Messages should be tailored to resonate with users’ sense of involvement and personal relevance while reducing psychological constraints. By aligning content strategies with both platform dynamics and users’ motivational states, health communicators can foster more effective engagement and ultimately promote meaningful behavioral change in response to public health challenges.

## Appendix

Multimedia Appendix 1 Descriptive statistics and analysis of measurement items.

## Abbreviations

AVE: average variance extracted
CAPS: communicative action in problem solving
CR: composite reliability
ILI: influenza-like illness
PLS-SEM: partial least squares structural equation modeling
STOPS: situational theory of problem solving
TAM: technology acceptance model
VIF: variance inflation factors

## Data Availability

Owing to ethical and legal restrictions, the datasets generated or analyzed during this study are not publicly available, but they are available from the corresponding author upon reasonable request.
