# Supplementary material for: Integrating the Situational Theory of Problem Solving and Technology Acceptance Model to Predict Intention to Practice Health Protective Behavior for Influenza-Like Illness Among TikTok Users: Cross-Sectional Study
Source: J Med Internet Res. 2025 Jul 2;27:e73677. doi: 10.2196/73677 (PMC12268223; doi:10.2196/73677)
Supplement: Multimedia Appendix 1 [file jmir_v27i1e73677_app1.doc]

| Second-order variable | Item | M (SD) | α | CR | factor loading | AVE |
| --- | --- | --- | --- | --- | --- | --- |
| First-order variable |  |  |  |  |  |  |
| Problem recognition (PR) |  | 4.32(0.32) | 0.718 | 0.725 |  | 0.548 |
|  | PR1: I feel surprised by influenza-like illness when they frequently appear on social media’s trending topics. |  |  |  | 0.594 |  |
|  | PR2: Influenza-like illnesses have happened in a way I did not expect. |  |  |  | 0.790 |  |
|  | PR3: I am very concerned about the influenza-like illness. |  |  |  | 0.818 |  |
|  | PR4: I think that influenza-like illness is a severe health problem. |  |  |  | 0.738 |  |
| Constraint recognition (CR) |  | 3.06(0.94) | 0.866 | 0.866 |  | 0.714 |
|  | CR1: I feel like my ideas and opinions matter to those in the government who are working on the influenza-like illness problem. |  |  |  | 0.831 |  |
|  | CR2: I feel that my opinions would be reflected in the policies and regulations related to the prevention and control of influenza-like illness. |  |  |  | 0.875 |  |
|  | CR3: I feel like my opinions could change public opinions on influenza-like illness. |  |  |  | 0.839 |  |
|  | CR4: If I were to propose suggestions for the prevention and control of influenza-like illness, there is a possibility that my suggestions could be adopted. |  |  |  | 0.834 |  |
| Involvement recognition (IR) |  | 4.48(0.79) | 0.767 | 0.776 |  | 0.587 |
|  | IR1: The influenza-like illness affects my life. |  |  |  | 0.796 |  |
|  | IR2: I am closely connected with influenza-like illness. |  |  |  | 0.752 |  |
|  | IR3: The influenza-like illnesses have serious consequences for me, my family, and friends. |  |  |  | 0.790 |  |
|  | IR4: If there is a large-scale outbreak of influenza-like illness, my life will be affected and changed. |  |  |  | 0.715 |  |
| Risk perception (RP) |  | 5.05(0.59) | 0.857 | 0.859 |  | 0.584 |
|  | RP1: How worried are you personally about the influenza-like illness issues at present? (1 = Not at All Worried, 6 = Very Worried |  |  |  | 0.736 |  |
|  | RP2: How likely do you think you will be directly and personally affected by influenza-like illness in the next months? (1 = Not at All Likely, 6 = Very Likely) |  |  |  | 0.71 |  |
|  | RP3: How likely do you think that your friends and family in the region you are currently living in will be directly affected by the influenza-like illness in the next months? (1 = Not at All Likely, 6 = Very Likely) |  |  |  | 0.723 |  |
|  | RP4: The influenza-like illness will affect very many people in the region I'm currently living in. |  |  |  | 0.667 |  |
|  | RP5: I will probably get sick with the influenza-like illness. |  |  |  | 0.664 |  |
|  | RP6: Getting sick with the influenza-like illness can be serious. |  |  |  | 0.74 |  |
| Situational motivation (SM) |  | 3.9(0.95) | 0.722 | 0.722 |  | 0.642 |
|  | SM1: I am curious about the influenza-like illness pandemic. |  |  |  | 0.801 |  |
|  | SM2: I frequently think about the influenza-like illness pandemic. |  |  |  | 0.824 |  |
|  | SM3: I would like to better understand the influenza-like illness pandemic. |  |  |  | 0.778 |  |
| Perceived usefulness (PU) |  | 4.50(0.83) | 0.805 | 0.806 |  | 0.719 |
|  | PU1: I think using TikTok would enhance my effectiveness in health management. |  |  |  | 0.848 |  |
|  | PU2: I think TikTok is useful for me to understand influenza-like illness. |  |  |  | 0.856 |  |
|  | PU3: I think TikTok is useful for me to make accurate decisions regarding health, such as wearing face masks. |  |  |  | 0.840 |  |
| Perceived ease of use (PEOU) |  | 4.65(0.81) | 0.728 | 0.752 |  | 0.648 |
|  | PEOU1: It is easy for me to learn using TikTok for influenza-like illness information. |  |  |  | 0.844 |  |
|  | PEOU2: I can easily become skillful at using TikTok for influenza-like illness information. |  |  |  | 0.856 |  |
|  | PEOU3: It would not require a lot of mental effort for me to interact with TikTok for influenza-like illness information. |  |  |  | 0.706 |  |
| Attitude toward using TikTok to obtain information about influenza-like illness (ATT) | “How would you rate TikTok? All things considered, using TikTok to obtain influenza-like illness information is…” | 4.55(0.90) | 0.947 | 0.947 |  | 0.703 |
|  | ATT1: Bad/Good (1 = Bad, 6 = Good) |  |  |  | 0.794 |  |
|  | ATT2: Foolish/Wise (1 = Foolish, 6 = Wise) |  |  |  | 0.833 |  |
|  | ATT3: Unfavorable/Favorable (1 = Unfavorable, 6 = Favorable) |  |  |  | 0.841 |  |
|  | ATT4: Harmful/Beneficial (1 = Harmful, 6 = Beneficial) |  |  |  | 0.850 |  |
|  | ATT5: Worthless/Valuable (1 = Worthless, 6 = Valuable) |  |  |  | 0.835 |  |
|  | ATT6: Negative/Positive (1 = Negative, 6 = Positive) |  |  |  | 0.856 |  |
|  | ATT7: Not helpful/Helpful (1 = Not helpful, 6 = Helpful) |  |  |  | 0.849 |  |
|  | ATT8: Unproductive/Productive (1 = Unproductive, 6 = Productive) |  |  |  | 0.822 |  |
|  | ATT9: Not useful/Useful (1 = Not useful, 6 = Useful) |  |  |  | 0.865 |  |
| Communicative action  in problem solving (CAPS) |  | 4.29(0.80) | 0.931 | 0.934 |  | 0.745 |
|  |  |  |  |  |  |  |
| Information seeking (ISK) |  | 4.20(0.99) | 0.871 | 0.871 |  | 0.722 |
|  | ISK1: I “like” or follow TikTok accounts for people or organizations related to the influenza-like illness. |  |  |  | 0.848 |  |
|  | ISK2： I use the TikTok to search for and subscribe to information about the influenza-like illness. |  |  |  | 0.867 |  |
|  | ISK3：I favorite certain influenza-like illness information on TikTok. |  |  |  | 0.860 |  |
|  | ISK4：I watch the relevant live streaming videos to get influenza-like illness information. |  |  |  | 0.823 |  |
| Information attending (IAT) |  | 4.21(0.92) | 0.847 | 0.852 |  | 0.686 |
|  | IAT1: While browsing TikTok, I randomly browse information that I regularly visit regarding influenza-like illness. |  |  |  | 0.774 |  |
|  | IAT2: I read other users’ comments on TikTok regarding influenza-like illness that I regularly visit. |  |  |  | 0.827 |  |
|  | IAT3: I click and follow hyperlinks on TikTok regarding influenza-like illness that I regularly visit. |  |  |  | 0.868 |  |
|  | IAT4: I subscribe to TikTok accounts (e.g., official media accounts, doctor’s personal accounts) and regularly read information regarding influenza-like illness. |  |  |  | 0.841 |  |
| Information forefending (IFF) |  | 4.76(0.76) | 0.750 | 0.771 |  | 0.667 |
|  | IFF1：I have invested enough time and energy into TikTok to understand the influenza-like illness. |  |  |  | 0.855 |  |
|  | IFF2：I can easily judge the value of information about the influenza-like illness on TikTok. |  |  |  | 0.850 |  |
|  | IFF3：I know where to go on TikTok when I need updated information regarding the influenza-like illness. |  |  |  | 0.739 |  |
| Information permitting (IPM) |  | 3.9(0.92) | 0.797 | 0.799 |  | 0.622 |
|  | IPM1: I welcome any information about influenza-like illness on TikTok. |  |  |  | 0.757 |  |
|  | IPM2: I am interested in all perspectives regarding influenza-like illnesses on TikTok. |  |  |  | 0.837 |  |
|  | IPM3: When discussing the issue of influenza-like illnesses on TikTok, I am patient and willing to listen to the opinions of those with whom I disagree. |  |  |  | 0.768 |  |
|  | IPM4: I am willing to pay attention to information on TikTok about influenza-like illnesses that conflicts with my own position. |  |  |  | 0.790 |  |
| Information forwarding (IFW) |  | 4.02(1.02) | 0.862 | 0.864 |  | 0.708 |
|  | IFW1：I am willing to spare my time to discuss influenza-like illness with someone I do not know well on TikTok |  |  |  | 0.828 |  |
|  | IFW2：When there are opportunities, I explain topics related to influenza-like illness to my family members and/or friends on TikTok. |  |  |  | 0.794 |  |
|  | IFW3：I (often) have conversations with friends about influenza-like illness on TikTok. |  |  |  | 0.871 |  |
|  | IFW4：I look for changes to share my knowledge and thoughts about the influenza-like illness on TikTok. |  |  |  | 0.871 |  |
| Information sharing (ISH) |  | 4.15(0.93) | 0.806 | 0.820 |  | 0.636 |
|  | ISH1: I post supportive or critical comments under certain information regarding influenza-like illness on TikTok. |  |  |  | 0.840 |  |
|  | ISH2: I share or retweet information about influenza-like illness that I discover by chance. |  |  |  | 0.827 |  |
|  | ISH3: When asked about my opinions regarding influenza-like illnesses, I am willing to share. |  |  |  | 0.669 |  |
|  | ISH4: I use TikTok to raise questions or make suggestions about the content that I see related to influenza-like illnesses. |  |  |  | 0.841 |  |
| Intention to practice health-  protective behaviors (BI) |  | 4.42(0.92) | 0.817 | 0.828 |  | 0.523 |
|  | How likely are you to engage in the following behaviors in the next month? (1 = Very Unlikely, 6 = Very Likely) |  |  |  |  |  |
|  | BI1: Wear a face mask in public. |  |  |  | 0.734 |  |
|  | BI2: Follow the prescribed 7 steps in hand washing. |  |  |  | 0.806 |  |
|  | BI3: Avoid people who cough in public without masks. |  |  |  | 0.667 |  |
|  | BI4: Avoid crowded places. |  |  |  | 0.760 |  |
|  | BI5: Adopt a balanced diet. |  |  |  | 0.638 |  |
|  | BI6: Take the influenza vaccine. |  |  |  | 0.720 |  |
